# The crosstalk between HIFs and mitochondrial dysfunctions in cancer development

**DOI:** 10.1038/s41419-021-03505-1

**Published:** 2021-02-26

**Authors:** Xingting Bao, Jinhua Zhang, Guomin Huang, Junfang Yan, Caipeng Xu, Zhihui Dou, Chao Sun, Hong Zhang

**Affiliations:** 1 Department of Medical Physics, Institute of Modern Physics, Chinese Academy of Sciences, Lanzhou, China; 2 Advanced Energy Science and Technology Guangdong Laboratory, Guangdong, China; 3 Key Laboratory of Heavy Ion Radiation Biology and Medicine of Chinese Academy of Sciences, Lanzhou, China; 4 College of Life Sciences, University of Chinese Academy of Sciences, Beijing, China; 5 School of Nuclear Science and Technology, University of Chinese Academy of Sciences, 101408 Beijing, China

**Keywords:** Cancer metabolism, Cancer microenvironment

## Abstract

Mitochondria are essential cellular organelles that are involved in regulating cellular energy, metabolism, survival, and proliferation. To some extent, cancer is a genetic and metabolic disease that is closely associated with mitochondrial dysfunction. Hypoxia-inducible factors (HIFs), which are major molecules that respond to hypoxia, play important roles in cancer development by participating in multiple processes, such as metabolism, proliferation, and angiogenesis. The Warburg phenomenon reflects a pseudo-hypoxic state that activates HIF-1α. In addition, a product of the Warburg effect, lactate, also induces HIF-1α. However, Warburg proposed that aerobic glycolysis occurs due to a defect in mitochondria. Moreover, both HIFs and mitochondrial dysfunction can lead to complex reprogramming of energy metabolism, including reduced mitochondrial oxidative metabolism, increased glucose uptake, and enhanced anaerobic glycolysis. Thus, there may be a connection between HIFs and mitochondrial dysfunction. In this review, we systematically discuss the crosstalk between HIFs and mitochondrial dysfunctions in cancer development. Above all, the stability and activity of HIFs are closely influenced by mitochondrial dysfunction related to tricarboxylic acid cycle, electron transport chain components, mitochondrial respiration, and mitochondrial-related proteins. Furthermore, activation of HIFs can lead to mitochondrial dysfunction by affecting multiple mitochondrial functions, including mitochondrial oxidative capacity, biogenesis, apoptosis, fission, and autophagy. In general, the regulation of tumorigenesis and development by HIFs and mitochondrial dysfunction are part of an extensive and cooperative network.

## Facts

- Mitochondrial dysfunction is closely related to different types of disease, including cancer.
- HIFs as major molecules that respond to hypoxia and regulate multiple processes such as metabolism, proliferation, and angiogenesis.
- The Warburg effect (aerobic glycolysis), a core hallmark of cancer cells, results in the activation of HIF-1α, and is related to mitochondrial dysfunction.
- Both HIFs and mitochondrial dysfunction can cause complex reprogramming of energy metabolism.

## Open questions

- Is there a relationship between HIFs and mitochondrial dysfunction in cancer development?

## Introduction

Mitochondria are essential cellular organelles that play important roles in regulating cellular energy, metabolism, survival, and proliferation^1,2^. Furthermore, dysfunction of mitochondria is closely associated with different types of diseases, including cancer^3^. Mitochondrial dysfunction of cancer cells includes enhancing glycolysis, reducing oxidative phosphorylation (OXPHOS), decreasing apoptosis, and increasing resistance to radiotherapy^4–6^. Mitochondrial dysfunction is also characterized by an inadequate number of mitochondria, aberrant mitochondrial morphology, dysfunction in electron transport, accumulation of mitochondrial reactive oxygen species (ROS), increased production of mitochondrial DNA (mtDNA) mutations, and oxidative damage to nucleic acids, proteins, and lipids^7–9^. Reprogramming of mitochondrial metabolism is a common sign of cancer. Abnormal energetic metabolism of cancer cells is known as the Warburg effect, which includes increased glucose uptake and high rates of glycolysis in combination with increased production of lactic acid, even under normoxic conditions^10–12^.

Hypoxia-inducible factors (HIFs) are major molecules that respond to hypoxia and regulate multiple processes such as metabolism, proliferation, and angiogenesis. All members are composed of two different subunits, including the α-subunit (HIF-1α, HIF-2α, or HIF-3α) and β-subunit (HIF-1β)^13,14^. Under hypoxic conditions, HIF-α combines with HIF-1β subunit to form a dimer, binds to hypoxia response elements (HREs), and causes expression of target genes^15,16^.

Many studies have established the role of HIFs in managing different signaling pathways in cancer, including cellular metabolism, cell proliferation and survival, angiogenesis, apoptosis, autophagy, extracellular matrix remodeling, and others^17,18^. The Warburg phenomenon reflects a pseudo-hypoxic state that activates HIF-1α^19,20^. The product of the Warburg effect, lactate, also induces HIF-1α and HIF-2α production^11,21,22^. However, Warburg proposed that aerobic glycolysis occurs due to a defect in the mitochondria^23^. More importantly, both HIFs and mitochondrial dysfunction can cause complex reprogramming of energy metabolism, including reduced mitochondrial oxidative metabolism, increased glucose uptake, and enhanced anaerobic glycolysis^4–6,18,24^. Thus, there is likely a connection between HIFs and mitochondrial dysfunction.

In fact, multiple studies have reported various relationships between HIFs and mitochondria. First, the stability and activity of HIFs are closely influenced by mitochondrial dysfunction related to the tricarboxylic acid (TCA) cycle, components of the electron transport chain (ETC), mitochondrial respiration, and mitochondria-related proteins. Furthermore, activation of HIFs can cause mitochondrial dysfunction by affecting numerous mitochondrial functions (i.e., mitochondrial oxidative capacity, biogenesis, apoptosis, fission, and autophagy). In this review, we systematically discuss the role of the crosstalk between HIFs and mitochondrial dysfunctions in cancer development.

## Role of mitochondrial dysfunction in cancer development

Mitochondria are essential organelles within the cell that regulate cellular energy, metabolism, survival, and proliferation. The mitochondria supply energy in the form of adenosine triphosphate (ATP), the synthesis of which is driven by a proton gradient^1,2,25^. Mitochondria are also recognized as a metabolic hub as the TCA cycle, which takes place within the mitochondria, coordinates the metabolism of carbohydrates, proteins, and fats into ATP^26^. Thus, with such a significant cellular role, dysfunction of the mitochondria has been shown to be related to various diseases, including cancer^3^. Mitochondrial dysfunction alters cellular energy metabolism, which contributes to carcinogenesis and tumor development^4,27,28^.

Mitochondrial dysfunction of cancer cells can include increasing glycolysis, reducing OXPHOS, decreasing apoptosis, and increasing resistance to radiotherapy^4–6^. In addition, mitochondrial dysfunction is often characterized by an inadequate number of mitochondria, aberrant mitochondrial morphology, dysfunction in electron transport, accumulation of mitochondrial ROS, increased production of mtDNA mutations, and oxidative damage to nucleic acids, proteins, and lipids^7,8^.

The reprogramming of mitochondrial metabolism is a common hallmark of cancer^10^. Cancer cells often switch metabolism from OXPHOS to aerobic glycolysis in order to produce energy, which can allow them to better adapt to the hypoxic tumor microenvironment and aid rapid proliferation^29,30^. The Warburg effect is associated with increased levels of glucose uptake and high rates of glycolysis, combined with the production of lactic acid, even in the presence of oxygen^11^. However, Warburg proposed that aerobic glycolysis occurs due to defects in mitochondria^23^. In addition, glycolysis is further enhanced by variation of mitochondrial function as well as aberrant accumulation of metabolites by affecting the nuclear genome through HIF-dependent pathways and histone modification^21^. The production of the Warburg effect and lactate also induces HIF-1α and HIF-2α^11,21,22^. HIF-1α, in turn, drives the expression of several glycolytic enzymes, including phosphofructokinase, glucose transporter-1, -3, hexokinase II (GLUT-1, -3), lactate dehydrogenase A (LDHA), and aldolase, which is involved in reprogramming aerobic glycolysis^31,32^. Moreover, the Warburg effect can be inhibited by targeting HIF-1α^33^.

## Role of HIFs in tumor progression

**Fig. 1:**
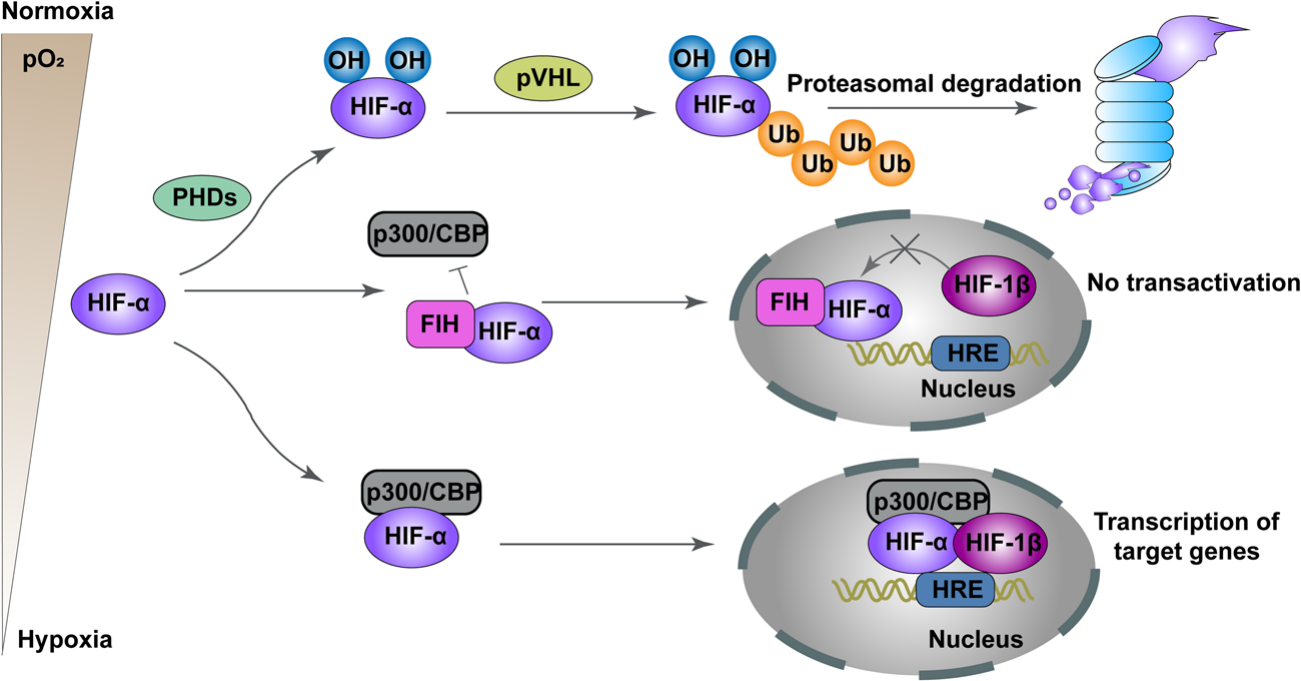
Schema of regulation of HIF-α degradation and transcriptional activity. Under conditions of normoxia, HIF-1α and HIF-2α are continuously degraded through the key oxygen sensor PHD1–3, especially PHD2 which enables HIF-α to bind to the pVHL. FIH also inhibits HIF-α by binding to HIF-α and impeding thecombination of HIF-α to the transcriptional coactivators CBP and p300. Under hypoxic conditions, the hydroxylation of HIFα is restrained, leading to stabilization of HIF-α. Next, HIF-α dimerizes with HIF-1β to comprise a transcriptional activation complex, which binds to HRE and stimulates the transactivation of target genes.

Production of HIFs is the major cellular response to hypoxia. All members of the HIF family are comprised of two different subunits, including an oxygen-labile α-subunit (HIF-1α, HIF-2α, or HIF-3α) and a constitutively expressed β-subunit (HIF-1β). HIF-1α is ubiquitously expressed across all tissues, while HIF-2α and HIF-3α are expressed in specific tissues^13,14^. While oxygen is available, HIF-1α and HIF-2α are constantly degraded by the key oxygen sensor prolyl hydroxylase (PHD)1–3, particularly PHD2 which enables HIF-α to bind to pVHL. Factor inhibiting HIF-1 (FIH-1) can also inhibit HIF-1α by binding to HIF-1α and inhibiting its transactivation^34,35^. However, under hypoxic conditions, PHD is inhibited, which allows HIF-α to accumulate and further dimerize with the HIF-1β subunit, bind to HRE, and lead to the activation of numerous genes (Fig. 1)^36–38^. However, HIF-3α plays a negative role in hypoxia-related gene expression, and overexpression of HIF-3α is associated with attenuation of angiogenesis and proliferation^39^.

**Fig. 2:**
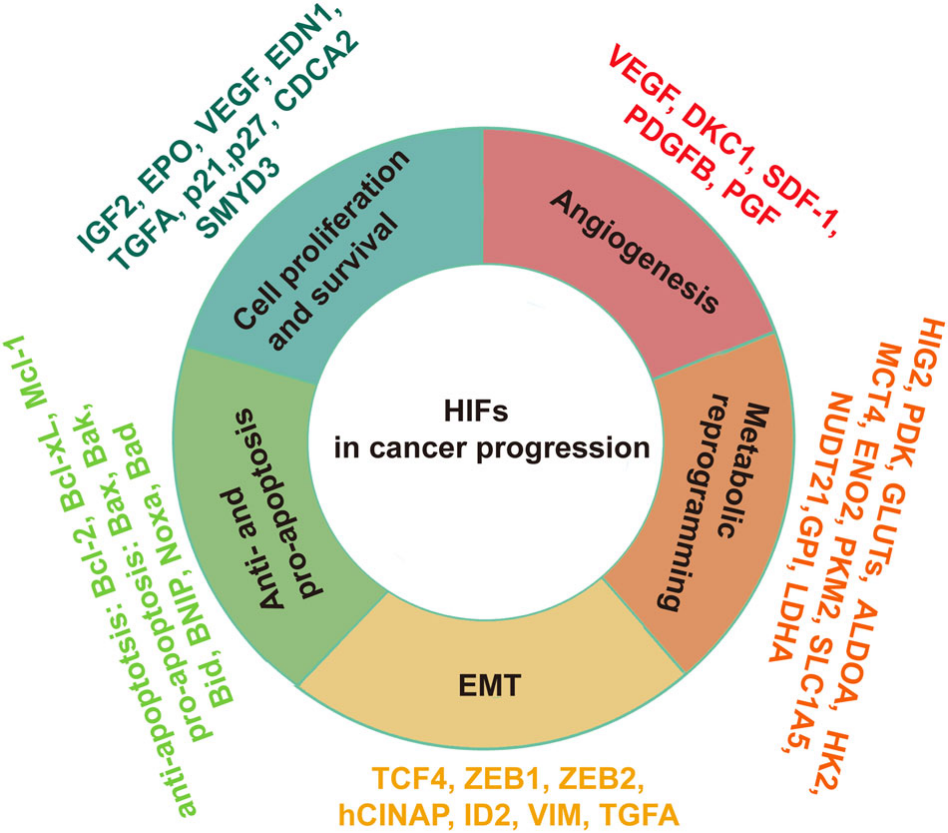
Role of HIFs in cancer progression. HIFs have a wide range of target genes which function to manage different types of signaling pathways in cancer. As an example, HIFs modulate cellular metabolic reprogramming, cell proliferation and survival, angiogenesis, apoptosis, and EMT.

Cancer cells frequently encounter hypoxia, and HIFs play a major role in the cellular mechanisms that are triggered in response to hypoxia^40^. Moreover, HIFs have a wide range of target genes that function to manage different types of signaling pathways in cancer (Fig. 2). As an example, HIFs modulate cellular metabolism, cell proliferation and survival, angiogenesis, apoptosis, autophagy, extracellular matrix remodeling, and additional tumor properties^17,18^.

### Reprogramming of energy metabolism

Cancer is both a genetic and metabolic disease due to mitochondrial dysfunction. Therefore, energy metabolism pathways are reprogrammed to meet the requirements of tumor cell proliferation and survival^41^. It has been shown that cancer cells prefer glycolysis as their energy source instead of OXPHOS, even in the presence of oxygen. Certain molecules like HIFs are essential to the survival of cancer cells in a hypoxic environment as transcriptional regulators of aerobic glycolysis^42,43^. The activation of HIFs in cancer can cause complex reprogramming of energy metabolism, including mitochondrial oxidative metabolism, glucose catabolism, glucose uptake, and energy production^18^. Activation of HIF-1 suppresses mitochondrial oxidative capacity by decreasing oxygen consumption, and preserves oxygen homeostasis in hypoxia. HIF-1 activation inhibits adipose triglyceride lipase-mediated lipolysis by hypoxia-inducible gene 2 (HIG2), leading to lipid droplet (LD) storage and declining mitochondrial fatty acid oxidation under hypoxia^44^. Furthermore, HIF-1α inhibits pyruvate conversion to acetyl-CoA by regulating pyruvate dehydrogenase kinase (PDK). Pyruvate plays a significant role in OXPHOS and mitochondrial electron transport^45^.

HIF-1α also regulates tumor growth by adjusting anaerobic and aerobic oxidation of glucose^46^. Glycolytic reprogramming is a key feature of metabolic reprogramming in tumors; thus, HIF-1α regulates glycolytic reprogramming by directly stimulating the transcription of all 12 enzymes that are necessary for glycolysis^47,48^. As an example, neuronal PAS domain protein 2 (NPAS2) upregulates glycolytic genes *GLUT1*, *HK2*, *ALDOA*, *GPI*, *MCT4*, *ENO2*, and *PKM2* by transcriptional upregulation of HIF-1α in hepatocellular carcinoma (HCC)^49^.

Moreover, HIF is related to glutamine metabolism in cancer development^50^. As an example, HIF-2α is involved in glutamine-induced ATP production by regulating the expression of *SLC1A5* variant in pancreatic cancer cells^51^. HIF-1α is also involved in glutamine metabolism by regulating NUDT21 in small cell lung cancer^52^.

### Cell proliferation and survival

The alterations in mitochondrial OXPHOS, energy production, glucose uptake and oxidation, and angiogenesis regulated by HIF-1 leads to enhanced cancer cell proliferation and survival^18^. The HIF pathway is involved in cancer cell proliferation through several molecular mechanisms. Vascular endothelial growth factor (*VEGF*), erythropoietin, insulin-like growth factor-2 (*IGF2*), transforming growth factor-α (*TGFA*), and endothelin 1 (*EDN1*) are particularly noteworthy target genes of the HIF pathway that are associated with aiding cell proliferation and survival. HIFs can alter cell cycle progression by directly targeting cyclin D1 and indirectly modulating p21 and p27^53–55^. In addition, the cell division cycle-associated protein (CDCA) family has vital functions in cell division and proliferation. CDCA2 promotes cell proliferation in prostate cancer and is known to be directly regulated by the HIF-1α pathway^56^. Pleomorphic adenoma gene like-2 (*PLAGL2*) has been shown to play an important role in tumorigenesis. In particular, the PLAGL2-EGFR-HIF-1/2α signaling loop has been reported to promote cellular proliferation in HCC^57^. SET and MYND domain-containing protein 3 (SMYD3) is a histone methyltransferase that is associated with gene transcription and oncogenesis. Depletion of *SMYD3* leads to an inhibition of renal cell carcinoma (RCC) cell proliferation, and HIF-2α can directly bind to the *SMYD3* promoter in order to stimulate *SMYD3* transcription and expression^58^. In Jak2V617F-positive myeloproliferative neoplasms, HIF-1 is required for cell growth and survival. Furthermore, suppression of HIF-1 binding to HREs by echinomycin causes damage to survival and growth of cancer cells by stimulating apoptosis and cell cycle arrest^59^.

### Angiogenesis

Inhibiting tumor angiogenesis by preventing the HIF-1α/VEGF/VEGFR-2 signaling pathway is believed to be a potential solid tumor-targeted therapy^60^. The HIF-1α/VEGF pathway is activated by multiple pathways^61^. Dyskeratosis congenita 1 is dysregulated across several cancers. In colorectal cancer (CRC), DKC1 stimulates angiogenesis and metastasis by stimulating HIF-1α and VEGF expression^62^. In addition to VEGF, HIFs also modulate angiogenic growth factor levels, including platelet-derived growth factor B, stromal-derived factor-1, and placenta growth factor^55^. However, HIF-3α hampers angiogenesis and proliferation by forming a complex with HIF-1α and preventing HIF transcription^63^.

### Apoptosis and autophagy

Studies conducted on the effect of HIF-1α on apoptosis have conflicting results. Many reports propose that HIF-1α can induce as well as antagonize apoptosis. HIF-1α is known to regulate both proapoptotic proteins (BNIP, Noxa, Bid, Bax, Bak, and Bad) and antiapoptotic proteins (Bcl-2, Bcl-xL, and Mcl-1)^64^. The Bcl-2/adenovirus E1B 19 kDa-interacting protein 3 (BNIP3) is a proapoptotic member of the Bcl-2 family that can be activated by HIF^65^. Although it has been strongly recommended that HIF-1α is an effective inducer of cellular apoptosis, recent studies indicate that apoptosis among colon cancer cells is enhanced by downregulating expression of HIF-1α and Slug with dictamnine^66^. Lung cancer apoptosis is also induced by inhibition of HIF-1α/VEGF signaling in A549 cells with tetrandrine^67^. The impact of HIF-2 on cellular apoptosis has been studied to a lesser extent, but points specifically to an antiapoptotic function^64^. For instance, HIF-2α knockdown promotes apoptosis and autophagy under hypoxic conditions in cervical cancer^68^.

Increasing evidence demonstrates that HIF-1 is directly involved in regulating mitochondrial autophagy by inducing expression of BH3-only proteins (BNIP3 and BNIP3L), which enables the release of Beclin-1, a significant regulator of autophagy^69^. The HIF-1α/BNIP3 signaling pathway has a significant function in activating hypoxia-induced autophagy in adenoid cystic carcinoma^70^. Furthermore, HIF-1α regulates hypoxia-stimulated autophagy by translocating ANKRD37, whose higher expression is correlated with decreased survival rates in colon cancer^71^. A novel HIF-1α/VMP1-autophagy pathway has also been reported in colon cancer cells^72^.

### Epithelial–mesenchymal transition (EMT)

EMT is a crucial regulator of cancer progression and metastasis^73^. HIFs are involved in EMT by the regulation of multiple pathways. HIF-1α inhibits E-cadherin expression by activating Snail, which facilitates EMT. In CRC cells, HIF-1α binds to β-catenin by competing with the transcription factor 4, which leads to induction of EMT. Moreover, HIF-1α enhances EMT and cancer metastasis by initiating the expression of zinc-finger E-box-binding homeobox 1 (ZEB1)^74^. In cervical cancer cells, HIF-1 induces EMT by binding to the human coilin-interacting nuclear ATPase protein (*hCINAP*) promoter and initiating expression of the gene under hypoxic conditions^75^. In HCC, thioredoxin-promoting EMT is HIF-2α-dependent^76^. In addition, ZEB2, inhibitor of differentiation 2, vimentin, and TGFA are all involved in EMT and are regulated by HIF expression^55^.

## Interplay between mitochondrial dysfunctions and HIFs

### Effects of mitochondrial dysfunction on HIFs

**Fig. 3:**
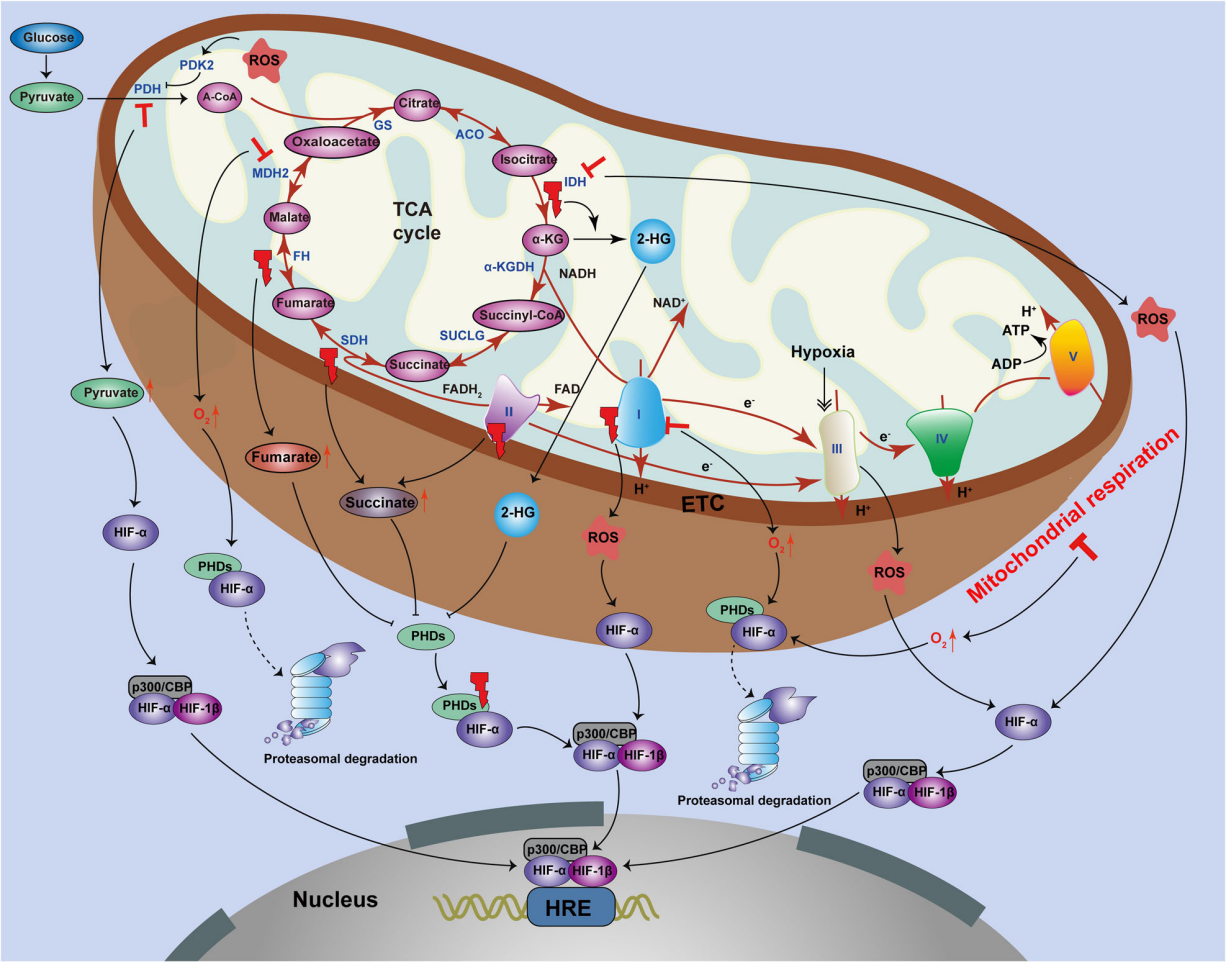
The effect of mitochondrial dysfunction on HIFs. The stability and activity of HIF-α are closely influenced by mitochondrial dysfunction related to the TCA cycle, ETC components, and mitochondrial respiration. First, dysregulation of the TCA cycle affects HIF stability and activity. Mutations of the TCA cycle enzymes, including IDH, SDH, and FH, cause stabilization and accumulation of HIF by inhibiting PHDs. Loss of IDH2 also leads to ROS-dependent stabilization of HIF-1α under normoxic conditions. However, inhibition of MDH2 stimulates HIF-1α degradation in cancer cells. Second, ETC components can have an effect on the stability and activity of HIFs. Mutations of complex I and complex II (also known as SDH) leads to HIF-1α stabilization by increasing ROS and succinate levels, respectively. The mitochondrial complex III can sense hypoxic conditions and produce ROS, which stabilizes the HIF-1α protein. However, deficiency and inhibition of complex I cause decreased HIF-1α stabilization using PHD-mediated degradation. In addition, suppression of mitochondrial respiration impedes stabilization of HIF-1α by reactivating PHD enzymes.

The stability and activity of HIFs are closely related to mitochondrial dysfunction (Fig. 3). First, dysregulation of the TCA cycle can have an effect on the stability and activity of HIFs. The mitochondrial TCA cycle forms a center in cellular metabolism due to the participation of multiple substrates, and disruption or deregulation of the TCA cycle enzymes can cause metabolic-mediated stabilization of HIF-1α by inhibiting PHDs^77,78^.

As an example, isocitrate dehydrogenase 2 (IDH2) is a TCA cycle enzyme that has been shown to be mutated in subsets of acute leukemias and gliomas. Loss of IDH2 in prostate cancer cells leads to ROS-dependent stabilization of HIF-1α underwent normoxic conditions, which is essential for increased mitochondrial trafficking and tumor cell movements^10^. IDH1 and IDH2 mutations fail to catalyze the conversion of isocitrate to α-ketoglutarate (α-KG), which leads to gaining de novo enzymatic activity. Eventually, this results in the reduction of α-KG to the metabolite 2-hydroxyglutarate (2-HG), which, in turn, inhibits PHDs, causing stabilization and accumulation of HIFs^79,80^.

Succinate dehydrogenase (SDH; also known as mitochondrial complex II), fumarate hydratase (FH), and malate dehydrogenase 2 (MDH2) are key TCA cycle enzymes, alterations of which have been shown to stimulate the activation of HIF signaling pathway. In pheochromocytomas (PHEOs) and paragangliomas (PGLs), SDH mutations cause high succinate accumulation. As a competitive inhibitor of PHDs, succinate leads to the activation of HIF-1α signaling pathway and the consequent expression of HIFs target genes. In the Hep3B hepatoma cell line, silencing of SDHB also stabilizes HIF-1α/2α and causes enrichment of functionally diverse genes, including hypoxia-related genes. Germline mutations of multiple subunits (SDHB/C/D) in RCC are associated with SDH-RCC hereditary cancer syndrome, a new type of aggressive kidney cancer. SDH mutations result in increased succinate levels, leading to the accumulation of HIFs in RCC. Inactivation or mutations of FH cause fumarate accumulation, and similar to succinate, fumarate causes HIF-1α activation by inhibition of PHD^81–85^. Mutations in SDH and FH have also been shown in patients with uterine and skin leiomyoma and papillary renal carcinoma. The consecutive accumulation of succinate and fumarate result in HIFs stabilization. In the SK-N-BE^2^ neuroblastoma cells, fumarate, but not succinate, has been shown to stabilize HIF-1α under normoxic conditions, while both fumarate and succinate induce HIF-2α^86^. Correspondingly, a reduction of transketolase in breast cancer cells leads to decreased HIF-1α by increasing levels of SDH, FH, and MDM2, causing inhibition of tumor metastasis^87^. MDH2 is a TCA cycle enzyme that is essential for energy production through respiration. Inhibition of MDH2 restrains mitochondrial respiration and causes a reduction in oxygen consumption, thus stimulating HIF-1α degradation in cancer cells^88,89^. As mentioned before, acid-enhanced production of L-2-HG leads to stabilization of HIF-1α under normoxic conditions. Furthermore, acid-enhanced conversion of α-KG to L-2-HG also can be stimulated by LDHA and MDH2. Thus, combined targeting of both LDHA and MDH2 abolishes L-2-HG production and reverses the ability of cells to stabilize HIF-1α^90^. Mitochondrial aconitase, the second enzyme of the TCA cycle, is involved in tumor development, which originates from the discovery that hypoxic conditions upregulate the HIF-1α target miR-210^83,91^.

Second, the stability and activity of HIFs can be influenced by components of the ETC. For instance, genetic ablation or pharmacologic inhibition of ETC components hinders stabilization of HIF-α in hypoxia^84^. In the CRC cell line, HCT116, and the osteosarcoma cell line, 143B, having a nuclear-encoded NDUFS3 knockout, where respiratory complex I deficiency leads to the PHD-mediated degradation of HIF-1α^92^. This was confirmed in another study^93^. A small inhibitor of complex I, AG311, has been shown to reduce HIF-1α stabilization by increasing oxygen tension in two breast cancer mouse models (MDA-MB-231 and MDA-MB-435). Furthermore, mutations of *MT-ND1*, *MT-ND2*, and *MT-ND5* genes, which encode components of complex I and play a role in OXPHOS, have been widely detected in various cancers. Both the *MT-ND1* (missense m.3460G > A, A52T) and *MT-ND2* (m.4776G > A, A103T) mutations are able to form tumors and represent a potential tumorigenic link to cytoplasmic ROS accumulation and HIF-1α stabilization. However, the osteosarcoma cybrids did not form tumors in vivo when the *MT-ND1*m.3571insC frameshift mutation induces accumulation of NADH, which, in turn, inhibits α-KG dehydrogenase, leading to increased α-KG and HIF-1α destabilization^2,94^.

SDH, an enzyme of the TCA, is also known as complex II and is comprised of four subunits: SDHA, SDHB, SDHC, and SDHD. SDH is responsible for producing mitochondrial energy and suppressing tumor activity. Mutations in SDH can cause succinate accumulation, which promotes glycolysis by stabilizing HIF-1α, as described earlier^95,96^. The R22X nonsense SDHD mutation of complex II has been shown to be present in hereditary PGL and PHEO, leading to a loss of complex II activities and succinate accumulation. Succinate has been shown to inhibit the activity of PHD, and it subsequently induces HIF-1α stabilization^2,5^. Mitochondrial complex III can sense hypoxic conditions and produce ROS, which stabilizes the HIF-1α protein. It has been reported that binding of terpestacin to the ubiquinol-cytochrome *c* reductase binding protein (UQCRB) subunit of complex III restrains HIF-1α stabilization by inhibiting ROS production, causing suppression of angiogenesis, which coincides with decreased VEGF levels^97^. Thus, the ETC components including complex I, complex II (SDH), and complex III play an important role in HIF stabilization^2,98^.

In addition, suppression of mitochondrial respiration causes oxygen redistribution from mitochondria to the cytoplasm, and obstructs stabilization of HIF-1α by reactivating the PHD enzymes^99^. Moreover, mutations in proteins that are involved in OXPHOS can also assist in increasing cellular ROS levels, which are regulated by catalase, glutathione peroxidase, and superoxide dismutase. The increased ROS production is known to upregulate HIF-1α expression by activating the PI3K/AKT signaling pathway. ROS production also activates PDK2, which suppresses pyruvate dehydrogenase (PDH) and leads to the accumulation of pyruvate, which can activate HIF-1α^2,100^. In triple-negative breast cancer (TNBC), MYC and MCL1 cooperate in order to maintain chemotherapeutic resistance of CSCs by increasing mitochondrial OXPHOS, leading to increased levels of ROS and accumulation of HIF-1α^101^. Furthermore, in CRC, inhibition of the c-Myc/ROS signaling pathway increases HIF-1α degradation, causing cell death under hypoxic conditions^102^.

Last, in addition to enzymes related to the TCA cycle, ETC components, and mitochondrial respiration, additional mitochondrial-related proteins can also have an effect on the stability and activity of HIFs. SIRT3, a member of Sirtuin (SIRT) family, is a major mitochondrial deacetylase. Reduced expression of SIRT3 in cancer cells stimulates ROS production, leading to HIF-1α stability and increased aerobic glycolysis. SIRT4, which plays a significant role in mitochondrial behaviors, is associated with increased ROS production, leading to stabilization of HIF-1α protein by hindering the catalytic activity of PHD^103^. Signal transducer and activator of transcription (STAT) proteins are known to be essential regulators of metabolism. To date, evidence exists that STAT3 and STAT5 can be found in mitochondria, and they influence the regulation of metabolic enzymes by mediating upregulation of HIF-1α expression. The constitutive activation of STAT3 induces HIF-1α expression, stimulates glycolysis, and decreases mitochondrial activity. Furthermore, STAT5 can induce HIF-2α expression^104^. In addition, the stability and activity of HIFs are associated with pVHL, casein kinase 2, and monoamine oxidase A, which are mitochondrial-related proteins^105–107^.

### Function of HIFs on mitochondrial dysfunction

**Fig. 4:**
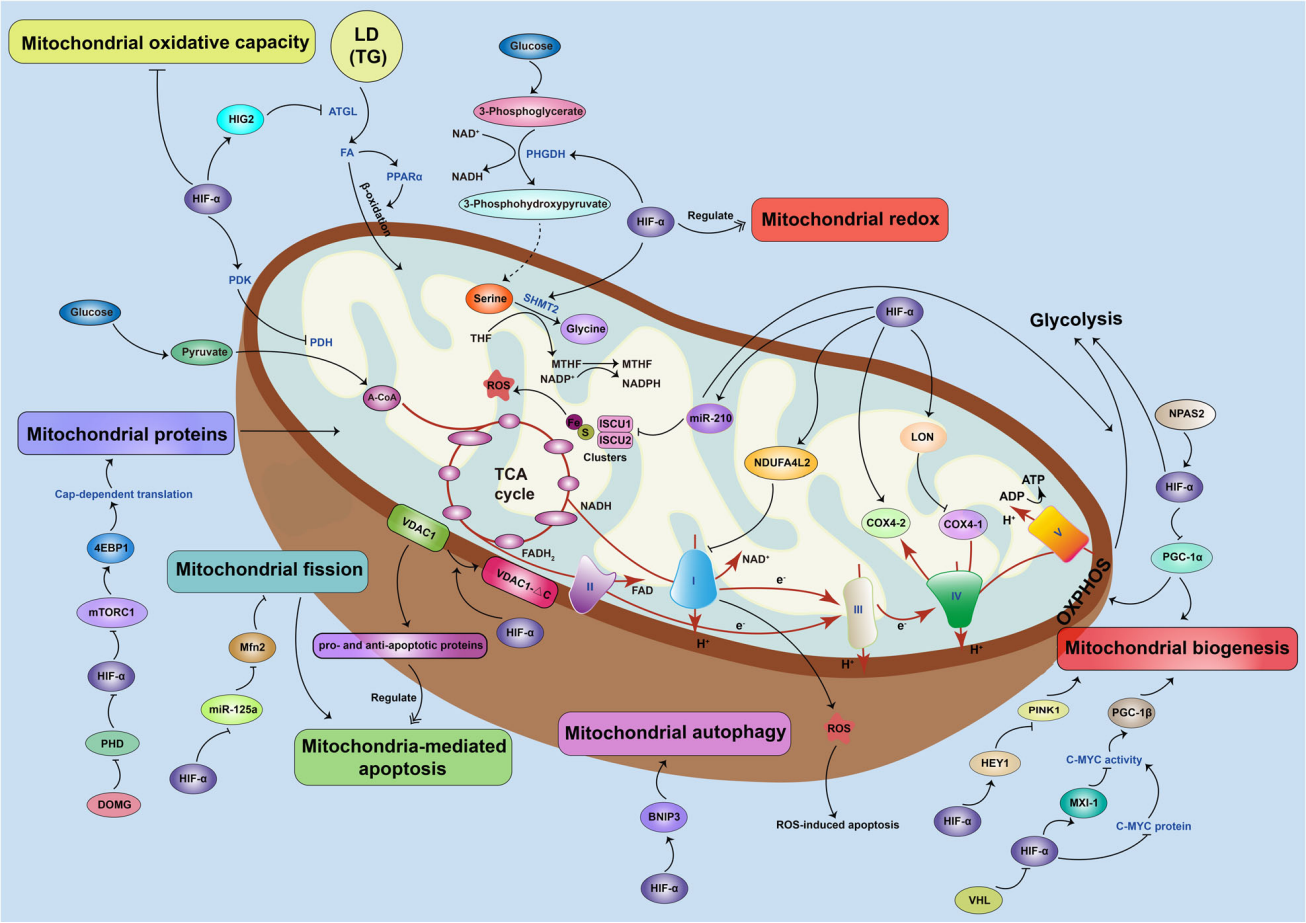
Function of HIFs on mitochondrial dysfunction. HIFs have an effect on multiple mitochondrial functions, including mitochondrial oxidative capacity, OXPHOS, biogenesis, apoptosis, fission, and autophagy. HIF-1 activation downregulates mitochondrial oxidation by inhibiting ATGL-mediated lipolysis via HIG2, which leads to LD storage and subsequently declines mitochondrial FA oxidation under hypoxic conditions, and by inducing PDK, which inhibits PDH, thereby inhibiting the flow of pyruvate into the TCA cycle. HIF-1 also regulates mitochondrial redox by inducing mitochondrial serine catabolism and the production of NADPH. Furthermore, miR-210, which is upregulated by HIF-1, restrains expression of the Fe–S cluster assembly proteins ISCU-1 and ISCU-2, which further restricts mitochondrial ROS generation and induces a shift of energy metabolism from OXPHOS to glycolysis. HIF-1 stimulates the less active ETC components, including NDUFA4L2, COX4-1, COX4-2, complex I, and complex IV, to delay the electron transfer through the ETC, therefore blocking the accumulation of ROS and reducing ROS-mediated apoptosis. HIF-1α reduces mitochondrial biogenesis by targeting PGC-1α, MXI, and HEY1. Meanwhile, NPAS2 increases the expression of glycolytic genes by transcriptionally upregulating HIF-1α. HIF-1 also regulates apoptosis by promoting the formation of VDAC1-∆C from VDAC1. Mitochondrial fission, which results in the activation of mitochondria-associated apoptosis, is negatively regulated by the HIF/miR-125a/Mfn2 pathways during carcinogenesis. Mitochondrial proteins can also be inhibited by suppression of the mTORC1/p70SK/S6 signaling pathway via action of HIF-1α. HIF-1α also induces BNIP3 to promote mitochondrial autophagy.

The activation of HIFs can cause mitochondrial dysfunction by affecting multiple mitochondrial activities, including mitochondrial oxidative capacity, biogenesis, apoptosis, fission, and autophagy, through various mechanisms (Fig. 4).

HIF-1 activation downregulates mitochondrial oxidative capacity by decreasing oxygen consumption and preserving oxygen homeostasis under hypoxic conditions. Earlier studies did not fully elucidate the mechanism by which HIF-1 functions to accelerate these two metabolic alterations. Recent studies have shown that HIF-1 activation inhibits adipose triglyceride lipase-mediated lipolysis by HIG2, leading to LD storage and declining mitochondrial fatty acid oxidation under hypoxic conditions^44^. Moreover, mitochondrial function and oxygen consumption are negatively regulated by HIF-1 by stimulating PDK, which inhibits PDH, and, consequently, blocks the flow of pyruvate into the TCA cycle^74^. If the inhibition of pyruvate flow is complete, the TCA cycle and OXPHOS would have to stop^108^. It has also been suggested that overexpression of HIF-1α contributes to inhibition of mitochondrial and oxidative damage induced by exposing antitumor drugs^109^. HIF-1α also stimulates catabolism of mitochondrial serine and NADPH production, which regulates mitochondrial redox by transactivation of serine hydroxymethyltransferase 2 and phosphoglycerate dehydrogenase (PHGDH), respectively. Reduced NADPH is used to maintain glutathione, the primary cellular antioxidant in a reduced situation. Furthermore, PHGDH-deficient breast cancer stem cells exhibit heightened oxidant levels and apoptosis in response to treatment with carboplatin or doxorubicin^110–112^.

Aberrant expression of miR-210 has been primarily correlated to the accumulation of HIF-1α. Meanwhile, it has been shown to be upregulated across various malignancies, including head and neck cancers, breast cancers, and pancreatic cancer. Furthermore, increased expression of miR-210 restrains the expression of the Fe–S cluster assembly proteins ISCU-1 and ISCU-2, which further restricts mitochondrial ROS generation^110,113^. It has been shown that miR-210 expression in proximal tubule cells may induce a shift of energetic metabolism from OXPHOS to glycolysis through the loss of mitochondrial inner membrane. Furthermore, a marked reduction of mitochondrial inner membrane and the metabolic shift towards glycolysis can imitate early events of clear cell renal cell carcinoma (ccRCC) development^113^. While promoting glycolytic activity, HIF-1 reduces mitochondrial activity by inducing activation of the less active ETC components, including NDUFA4L2, COX4-2, complex I, and complex IV, in order to delay electron transfer through the ETC. Hence, the process blocks the accumulation of ROS and reduces ROS-mediated apoptosis. In addition, HIF-1α prompts the expression of mitochondrial LON peptidase, which leads to degradation of the mitochondrial protein COX4-1 in order to decrease mitochondrial flux under hypoxic conditions^114^. It has also been demonstrated that HIF-1α is able to prevent the metabolism of coenzyme A (CoA) in mitochondria, as well as mitochondrial biogenesis^115^.

NPAS2, a critical oncogene in hepatocellular carcinogenesis, plays an important role in HCC tumor progression. It has been demonstrated that NPAS2 inhibits mitochondrial biogenesis and OXPHOS by downregulating peroxisome proliferator-activated receptor gamma coactivator-1 α (PGC-1α) by transcriptionally upregulating HIF-1α in HCC cells. Furthermore, PGC-1α also upregulates glycolytic genes through the transcriptional activation of HIF-1α^49^. In addition, HIF-1 reduces mitochondrial biogenesis by targeting MAX interactor 1, which restrains C-MYC-mediated transcription of the peroxisome PGC-1β. Apart from these, it has been shown that HIF decreases mitochondrial biogenesis by increasing HEY1 and transcriptionally repressing PTEN-induced putative kinase 1^116^.

The voltage-dependent anion channel 1 (VDAC1) is a protein present in the outer mitochondria membrane (OMM) that mediates the transport of nucleotides, Ca^2+^, and additional metabolites via the OMM. VDAC1 also modulates mitochondria-mediated apoptosis by inducing the release of pro- and antiapoptotic proteins. Under hypoxic conditions, VDAC1 is truncated at the C terminus (VDAC1-∆C). Studies have demonstrated that VDAC1-∆C is highly expressed at the advanced stage of lung cancer. Furthermore, under hypoxic conditions, HIF-1α expression activates a cascade of events that lead to VDAC1-∆C formation in HeLa cells^117^. In pancreatic cancer cell line PANC-1, studies have shown that mitochondrial fission (involved in apoptosis, migration, and energy metabolism) is regulated by the HIF/miR-125a/Mfn2 pathways during pancreatic carcinogenesis. Furthermore, miR-125a, which is negatively regulated by HIF-1, promotes cancer cell-mediated mitochondrial death by inducing mitochondrial fission, which leads to the activation of mitochondria-associated apoptotic pathways^118^.

Moreover, the expression of mitochondrial proteins can be inhibited by dimethyloxaloylglycine, a PHD inhibitor, that results from the suppression of the mTORC1/p70SK/S6 signaling pathway through HIF-1α^119^. During hypoxia, HIF-1 induces BNIP3 to promote mitochondrial autophagy in order to contribute to chemoresistance and facilitate cell survival by cooperating with BECLIN-1 and ATG544^110,120^.

### Targeting HIF or mitochondria in cancer

Small-molecule inhibitors of HIFs have been identified as inhibiting numerous activities, including inhibition of HIF messenger RNA (mRNA) expression, HIF protein synthesis, transcriptional activities of HIF, a combination of HIF with its coactivators, heterodimerization of HIF-α and HIF-β, and the HREs-DNA binding. Some of the small-molecule inhibitors have been studied in a phase II or III clinical trial, including 2-methoxyestradiol (2ME2), tanespimycin, vorinostat, PT2385, PT2977, and CRLX101^13,121^. Inhibition of HIFs can have a profound effect on mitochondrial function and can affect numerous processes such as mitochondrial OXPHOS, ROS accumulation, lipid peroxidation, and ATP generation. 2ME2, an inhibitor of HIFs, has been identified as a novel anticancer agent. In acute myeloid leukemia, 2ME2 increases ROS generation, and stimulates the mitochondrial apoptotic pathway by inhibiting HIF-1α expression^14^. Vosaroxin, a quinolone-derivative anticancer agent, inhibits HIF-1α protein synthesis and impedes the dimerization of HIF-1α and HIF-1β. Vosaroxin significantly increases levels of mitochondrial ROS and lipid peroxidation, and induces mitochondrial swelling and ATP generation by acting through the AMPK/Sirt3/HIF-1 pathway in the cervical cancer cell line HeLa^122^. Cardamonin, a chalcone isolated from *Alpiniae katsumadai*, suppresses HIF-1α expression at both the mRNA and protein levels by impeding the mTOR/p70S6K pathway. Cardamonin inhibits the growth of the TNBC MDA-MB-231 cells by suppressing HIF-1α. Subsequently, it enhances mitochondrial OXPHOS and induces ROS accumulation^123^. NDUFA4L2, a less active complex I subunit within the ETC, is significantly overexpressed in HCC and other human cancers. Furthermore, NDUFA4L2 is HIF-1-regulated in HCC cells. Mitochondrial activity and oxygen consumption are also increased by inhibition of the HIF-1/NDUFA4L2 pathway, which results in ROS accumulation and apoptosis^114^.

In addition, HIF-1α is known to be a contributor to resistance to chemotherapy and radiation. In fact, many mechanisms involved in the activation of the HIF-1α-mediated DNA repair pathway, metabolic reprogramming, apoptotic inhibition, and autophagy activation have roles in HIF-1α-mediated chemo-/radioresistance^124^. Thus, HIF-1α can be targeted to overcome chemo-/radioresistance. HIF-1α has a central role in resistance of pancreatic ductal adenocarcinoma towards chemotherapy and radiotherapy. Inhibition of HSP90, a key chaperone protein of HIF-1α, overcomes resistance to chemotherapy and radiotherapy in pancreatic cancer^125^. Bortezomib, a reversible proteasome inhibitor, sensitizes esophageal squamous cancer cells to radiotherapy by decreasing HIF-1α and VEGF expression^126^.

Mitochondria-targeting drugs are also a promising and effective strategy for cancer treatment^127,128^. Likewise, targeting mitochondrial function can also help alter HIF expression. AG311, a small anticancer molecule, has been shown to competitively inhibit complex I activity. HIF-1α stabilization is decreased by inhibition of mitochondrial oxygen consumption with AG311 by increasing oxygen tension under hypoxic conditions^93^. The ccRCC demonstrates inhibition of mitochondrial function and preferential use of glycolysis, even under normoxic conditions. Dichloroacetate, the PDK inhibitor, reactivates mitochondrial function, which includes enhancing respiration and levels of TCA metabolites (i.e., α-KG), and subsequently reduces HIF transcriptional activity in an FIH-dependent manner. FIH is associated with mitochondrial function as it requires α-KG as a cofactor^129^. The UQCRB of mitochondrial complex III is a novel therapeutic target for cancer treatment. UQCRB inhibitors regulate mitochondrial function in glioblastoma stem-like cells by decreasing mitochondrial ROS generation, as well as the mitochondrial membrane potential. The inhibition of mitochondrial ROS generation through the use of UQCRB inhibitors block HIF activation^130^.

## Conclusions

An ever-increasing number of studies show that tumors are likely a complex disease associated with impairment of energetic metabolism. Cancer cells depend on metabolic adaptations in order to preserve energy production, support cell growth, and produce signaling molecules for various tumor-promoting activities. Therefore, it is essential to do an in-depth study of the process of metabolic adaptation in order to ascertain weaknesses of tumor metabolic pathways and establish an effective treatment strategy. Both HIFs and mitochondrial dysfunction are important regulatory factors that play a role in metabolic adaptations of cancer cells. Both of these mechanisms cause complex reprogramming of energy metabolism, including reduced mitochondrial oxidative metabolism, increased glucose uptake, and enhanced anaerobic glycolysis. Furthermore, dynamic changes to the response and use of oxygen by tumor cells are the source of abnormal energy metabolism in tumors. HIF and mitochondria are also two central weapons for tumor cells to cope with changes in oxygen dynamics. Despite the fact that more evidence is needed to prove our hypothesis, this review reveals that a relationship between HIFs and mitochondria can actively promote a new understanding of tumor occurrence and development, and provide a novel entry point for the formulation of tumor prevention and treatment programs.

Numerous scientific studies have illustrated that the stability and activity of HIFs are closely influenced by mitochondrial dysfunction related to the TCA cycle, ETC components, mitochondrial respiration, and mitochondrial-related proteins. In addition, activation of HIFs can cause mitochondrial dysfunction by influencing multiple mitochondrial functions, including mitochondrial oxidative capacity, biogenesis, apoptosis, fission, and autophagy. In addition, targeting HIFs can not only affect mitochondria function but mitochondria-targeting drug can also affect the stability and activity of HIFs. In general, the regulation of tumorigenesis and development by HIFs and mitochondrial dysfunction are part of an extensive and cooperative network. However, the current studies have only investigated the regulation of HIFs or mitochondrial dysfunction from a single aspect, and have ignored their collaboration in regulating carcinogenesis and progression. Future research is needed to expose the full spectrum of interplay between HIFs and mitochondrial dysfunction in specific tumor settings, and to reveal how they synergistically influence the oxygen dynamics in cancer development. Novel insight into this process will likely open up additional diagnostic and therapeutic approaches that can help improve outcomes for patients with tumors.

## References

[1] Momcilovic M (2019). In vivo imaging of mitochondrial membrane potential in non-small-cell lung cancer. Nature.

[2] Nakhle J, Rodriguez A-M, Vignais M-L (2020). Multifaceted roles of mitochondrial components and metabolites in metabolic diseases and cancer. Int. J. Mol. Sci..

[3] Han Y (2019). Mitochondrial fission causes cisplatin resistance under hypoxic conditions via ROS in ovarian cancer cells. Oncogene.

[4] Sun C (2019). Endocytosis-mediated mitochondrial transplantation: transferring normal human astrocytic mitochondria into glioma cells rescues aerobic respiration and enhances radiosensitivity. Theranostics.

[5] Yang J (2019). The enhancement of glycolysis regulates pancreatic cancer metastasis. Cell. Mol. Life Sci..

[6] Deng P, Haynes CM (2017). Mitochondrial dysfunction in cancer: potential roles of ATF5 and the mitochondrial UPR. Semin. Cancer Biol..

[7] Chen G, Kroemer G, Kepp O (2020). Mitophagy: an emerging role in aging and age-associated diseases. Front. Cell Dev. Biol..

[8] Sekar D (2020). Biological and clinical relevance of microRNAs in mitochondrial diseases/dysfunctions. DNA Cell Biol..

[9] Chen, K. et al. Mitochondrial mutations and mitoepigenetics: focus on regulation of oxidative stress-induced responses in breast cancers. *Semin. Cancer Biol.*10.1016/j.semcancer.2020.09.012 (2020). 10.1016/j.semcancer.2020.09.012 33035656

[10] Wang Y (2019). IDH2 reprograms mitochondrial dynamics in cancer through a HIF-1α-regulated pseudohypoxic state. FASEB J..

[11] Barbosa AM, Martel F (2020). Targeting glucose transporters for breast cancer therapy: the effect of natural and synthetic compounds. Cancers.

[12] Nie H (2020). O-GlcNAcylation of PGK1 coordinates glycolysis and TCA cycle to promote tumor growth. Nat. Commun..

[13] Tang W, Zhao G (2020). Small molecules targeting HIF-1alpha pathway for cancer therapy in recent years. Bioorg. Med. Chem..

[14] Zhe N (2016). HIF-1alpha inhibition by 2-methoxyestradiol induces cell death via activation of the mitochondrial apoptotic pathway in acute myeloid leukemia. Cancer Biol. Ther..

[15] Wu D, Potluri N, Lu J, Kim Y, Rastinejad F (2015). Structural integration in hypoxia-inducible factors. Nature.

[16] Barben M (2018). Hif1α inactivation rescues photoreceptor degeneration induced by a chronic hypoxia-like stress. Cell Death Differ..

[17] Guo Y (2020). Hypoxiainducible factors in hepatocellular carcinoma (Review). Oncol. Rep..

[18] Moldogazieva NT, Mokhosoev IM, Terentiev AA (2020). Metabolic heterogeneity of cancer cells: an interplay between HIF-1, GLUTs, and AMPK. Cancers.

[19] Dasgupta A (2020). Mitochondria in the pulmonary vasculature in health and disease: oxygen-sensing, metabolism, and dynamics. Compr. Physiol..

[20] Hayashi Y, Yokota A, Harada H, Huang G (2019). Hypoxia/pseudohypoxia‐mediated activation of hypoxia‐inducible factor‐1α in cancer. Cancer Sci..

[21] Jia D, Park JH, Jung KH, Levine H, Kaipparettu BA (2018). Elucidating the metabolic plasticity of cancer: mitochondrial reprogramming and hybrid metabolic states. Cells.

[22] Perez-Escuredo J (2016). Lactate promotes glutamine uptake and metabolism in oxidative cancer cells. Cell Cycle.

[23] Warburg O (1956). On the origin of cancer cells. Science.

[24] Li X (2019). Hypoxia-induced autophagy of stellate cells inhibits expression and secretion of lumican into microenvironment of pancreatic ductal adenocarcinoma. Cell Death Differ..

[25] Sharma A (2018). Targeting mitochondrial dysfunction and oxidative stress in activated microglia using dendrimer-based therapeutics. Theranostics.

[26] Noe JT, Mitchell RA (2019). Tricarboxylic acid cycle metabolites in the control of macrophage activation and effector phenotypes. J. Leukoc. Biol..

[27] Li N, Zhan X (2019). Mitochondrial dysfunction pathway networks and mitochondrial dynamics in the pathogenesis of pituitary adenomas. Front. Endocrinol..

[28] Dong Z, Pu L, Cui H (2020). Mitoepigenetics and its emerging roles in cancer. Front. Cell Dev. Biol..

[29] Mascaraque M (2020). Metformin as an adjuvant to photodynamic therapy in resistant basal cell carcinoma cells. Cancers.

[30] Whitehall JC, Greaves LC (2020). Aberrant mitochondrial function in ageing and cancer. Biogerontology.

[31] Wu Z, Wu J, Zhao Q, Fu S, Jin J (2020). Emerging roles of aerobic glycolysis in breast cancer. Clin. Transl. Oncol..

[32] Gao T (2020). SIK2 promotes reprogramming of glucose metabolism through PI3K/AKT/HIF-1α pathway and Drp1-mediated mitochondrial fission in ovarian cancer. Cancer Lett..

[33] Lu J (2020). Ginsenoside 20(S)-Rg3 upregulates HIF-1alpha-targeting miR-519a-5p to inhibit the Warburg effect in ovarian cancer cells. Clin. Exp. Pharmacol. Physiol..

[34] Li H, Jia Y, Wang Y (2019). Targeting HIF-1alpha signaling pathway for gastric cancer treatment. Die Pharmazie.

[35] Qi Y (2015). PTEN induces apoptosis and cavitation via HIF-2-dependent Bnip3 upregulation during epithelial lumen formation. Cell Death Differ..

[36] Li A (2019). The roles and signaling pathways of prolyl-4-hydroxylase 2 in the tumor microenvironment. Chem. Biol. Interact..

[37] Wang X (2020). A novel LncRNA HITT forms a regulatory loop with HIF-1alpha to modulate angiogenesis and tumor growth. Cell Death Differ..

[38] Jain CV (2017). Trophoblast survival signaling during human placentation requires HSP70 activation of MMP2-mediated HBEGF shedding. Cell Death Differ..

[39] Barth DA (2020). Long-noncoding RNA (lncRNA) in the regulation of hypoxia-inducible factor (HIF) in cancer. Non-Coding RNA.

[40] Jing X (2019). Role of hypoxia in cancer therapy by regulating the tumor microenvironment. Mol. Cancer.

[41] Zam, W., Ahmed, I. & Yousef, H. Warburg effects on cancer cells survival: the role of sugar starvation in cancer therapy. *Curr. Clin. Pharmacol.*10.2174/1574884715666200413121756 (2020). 10.2174/1574884715666200413121756 32282309

[42] Du Y, Wei N, Ma R, Jiang S, Song D (2020). A miR-210-3p regulon that controls the Warburg effect by modulating HIF-1alpha and p53 activity in triple-negative breast cancer. Cell Death Dis..

[43] Cao L (2020). Circular RNA circRNF20 promotes breast cancer tumorigenesis and Warburg effect through miR-487a/HIF-1alpha/HK2. Cell Death Dis..

[44] Zhang X (2017). Inhibition of intracellular lipolysis promotes human cancer cell adaptation to hypoxia. eLife.

[45] Duan J (2020). Phenolic compound ellagic acid inhibits mitochondrial respiration and tumor growth in lung cancer. Food Funct..

[46] Huang M (2020). Autonomous glucose metabolic reprogramming of tumour cells under hypoxia: opportunities for targeted therapy. J. Exp. Clin. Cancer Res..

[47] Becker LM (2020). Epigenetic reprogramming of cancer-associated fibroblasts deregulates glucose metabolism and facilitates progression of breast cancer. Cell Rep..

[48] Heydarzadeh S, Moshtaghie AA, Daneshpoor M, Hedayati M (2020). Regulators of glucose uptake in thyroid cancer cell lines. Cell Commun. Signal..

[49] Yuan P (2020). Circadian clock gene NPAS2 promotes reprogramming of glucose metabolism in hepatocellular carcinoma cells. Cancer Lett..

[50] Povero D, Johnson SM, Liu J (2020). Hypoxia, hypoxia-inducible gene 2 (HIG2)/HILPDA, and intracellular lipolysis in cancer. Cancer Lett..

[51] Yoo HC (2020). A variant of SLC1A5 is a mitochondrial glutamine transporter for metabolic reprogramming in cancer cells. Cell Metab..

[52] Gao CC (2020). NUDT21 suppresses the growth of small cell lung cancer by modulating GLS1 splicing. Biochem. Biophys. Res. Commun..

[53] Kumar H, Choi DK (2015). Hypoxia inducible factor pathway and physiological adaptation: a cell survival pathway?. Mediat. Inflamm..

[54] Courtnay R (2015). Cancer metabolism and the Warburg effect: the role of HIF-1 and PI3K. Mol. Biol. Rep..

[55] Akanji MA, Rotimi D, Adeyemi OS (2019). Hypoxia-inducible factors as an alternative source of treatment strategy for cancer. Oxid. Med. Cell. Longev..

[56] Zhang Y (2020). CDCA2 inhibits apoptosis and promotes cell proliferation in prostate cancer and is directly regulated by HIF-1alpha pathway. Front. Oncol..

[57] Hu, W. et al. PLAGL2-EGFR-HIF-1/2alpha signaling loop promotes HCC progression and Erlotinib insensitivity. *Hepatology* **73**, 674–691 (2020). 10.1002/hep.31293 32335942

[58] Liu C (2020). VHL-HIF-2alpha axis-induced SMYD3 upregulation drives renal cell carcinoma progression via direct trans-activation of EGFR. Oncogene.

[59] Baumeister J (2019). Hypoxia-inducible factor 1 (HIF-1) is a new therapeutic target in JAK2V617F-positive myeloproliferative neoplasms. Leukemia.

[60] Zhang P-C (2020). AT-533, a novel Hsp90 inhibitor, inhibits breast cancer growth and HIF-1α/VEGF/VEGFR-2-mediated angiogenesis in vitro and in vivo. Biochem. Pharmacol..

[61] Ndiaye PD (2019). VEGFC acts as a double-edged sword in renal cell carcinoma aggressiveness. Theranostics.

[62] Hou P (2020). DKC1 enhances angiogenesis by promoting HIF-1alpha transcription and facilitates metastasis in colorectal cancer. Br. J. Cancer.

[63] Bowler, E. & Oltean, S. Alternative splicing in angiogenesis. *Int. J. Mol. Sci*. **20**, 2067–2094 (2019). 10.3390/ijms20092067 PMC6540211 31027366

[64] Sendoel A, Hengartner MO (2014). Apoptotic cell death under hypoxia. Physiology.

[65] Shao Y (2018). Expression and epigenetic regulatory mechanism of BNIP3 in clear cell renal cell carcinoma. Int. J. Oncol..

[66] Wang JY (2018). Dictamnine promotes apoptosis and inhibits epithelial-mesenchymal transition, migration, invasion and proliferation by downregulating the HIF-1alpha and Slug signaling pathways. Chem. Biol. Interact..

[67] Chen Z, Zhao L, Zhao F, Yang G, Wang JJ (2018). Tetrandrine suppresses lung cancer growth and induces apoptosis, potentially via the VEGF/HIF-1alpha/ICAM-1 signaling pathway. Oncol. Lett..

[68] Jiang L (2018). MicroRNA-519d-3p inhibits proliferation and promotes apoptosis by targeting HIF-2alpha in cervical cancer under hypoxic conditions. Oncol. Res..

[69] Wigerup C, Pahlman S, Bexell D (2016). Therapeutic targeting of hypoxia and hypoxia-inducible factors in cancer. Pharmacol. Ther..

[70] Wu H (2015). Hypoxia-induced autophagy contributes to the invasion of salivary adenoid cystic carcinoma through the HIF-1alpha/BNIP3 signaling pathway. Mol. Med. Rep..

[71] Deng M, Zhang W, Yuan L, Tan J, Chen Z (2020). HIF-1a regulates hypoxia-induced autophagy via translocation of ANKRD37 in colon cancer. Exp. Cell Res..

[72] Rodriguez ME, Catrinacio C, Ropolo A, Rivarola VA, Vaccaro MI (2017). A novel HIF-1alpha/VMP1-autophagic pathway induces resistance to photodynamic therapy in colon cancer cells. Photochem. Photobiol. Sci..

[73] Wang M, Yan J, Cao X, Hua P, Li Z (2020). Hydrogen sulfide modulates epithelial-mesenchymal transition and angiogenesis in non-small cell lung cancer via HIF-1α activation. Biochem. Pharmacol..

[74] Lee SY (2018). Oncogenic metabolism acts as a prerequisite step for induction of cancer metastasis and cancer stem cell phenotype. Oxid. Med. Cell. Longev..

[75] Zhang, Y. et al. hCINAP is a potential direct HIF-1 target gene and is required for hypoxia-induced EMT and apoptosis in cervical cancer cells. *Biochem. Cell Biol.*10.1139/bcb-2020-0090 (2020). 10.1139/bcb-2020-0090 32830518

[76] Cao MQ (2020). Cross talk between oxidative stress and hypoxia via thioredoxin and HIF-2alpha drives metastasis of hepatocellular carcinoma. FASEB J..

[77] Martinez-Reyes I, Chandel NS (2020). Mitochondrial TCA cycle metabolites control physiology and disease. Nat. Commun..

[78] Paredes F (2018). Poldip2 is an oxygen-sensitive protein that controls PDH and alphaKGDH lipoylation and activation to support metabolic adaptation in hypoxia and cancer. Proc. Natl Acad. Sci. USA.

[79] Semukunzi H (2017). IDH mutations associated impact on related cancer epidemiology and subsequent effect toward HIF-1alpha. Biomed. Pharmacother..

[80] Heuser M, Araujo Cruz MM, Goparaju R, Chaturvedi A (2015). Enigmas of IDH mutations in hematology/oncology. Exp. Hematol..

[81] Jochmanova I, Zhuang Z, Pacak K (2015). Pheochromocytoma: gasping for air. Hormones Cancer.

[82] Yao J (2018). Combinatorial treatment of Rhizoma Paridis saponins and sorafenib overcomes the intolerance of sorafenib. J. Steroid Biochem. Mol. Biol..

[83] Desideri E, Vegliante R, Ciriolo MR (2015). Mitochondrial dysfunctions in cancer: genetic defects and oncogenic signaling impinging on TCA cycle activity. Cancer Lett..

[84] Sharma S, Wang J, Cortes Gomez E, Taggart RT, Baysal BE (2017). Mitochondrial complex II regulates a distinct oxygen sensing mechanism in monocytes. Hum. Mol. Genet..

[85] Ciccarese C (2016). The prospect of precision therapy for renal cell carcinoma. Cancer Treat. Rev..

[86] Laukka T (2016). Fumarate and succinate regulate expression of hypoxia-inducible genes via TET enzymes. J. Biol. Chem..

[87] Tseng CW (2018). Transketolase regulates the metabolic switch to control breast cancer cell metastasis via the alpha-ketoglutarate signaling pathway. Cancer Res..

[88] Naik R (2017). Methyl 3-(3-(4-(2,4,4-Trimethylpentan-2-yl)phenoxy)-propanamido)benzoate as a novel and dual malate dehydrogenase (MDH) 1/2 inhibitor targeting cancer metabolism. J. Med. Chem..

[89] Ban HS (2016). A novel malate dehydrogenase 2 inhibitor suppresses hypoxia-inducible factor-1 by regulating mitochondrial respiration. PLoS ONE.

[90] Intlekofer AM (2017). L-2-hydroxyglutarate production arises from noncanonical enzyme function at acidic pH. Nat. Chem. Biol..

[91] Sharkia R (2019). Clinical, radiological, and genetic characteristics of 16 patients with ACO2 gene defects: delineation of an emerging neurometabolic syndrome. J. Inherit. Metab. Dis..

[92] Kurelac I (2019). Inducing cancer indolence by targeting mitochondrial complex I is potentiated by blocking macrophage-mediated adaptive responses. Nat. Commun..

[93] Bastian A (2017). AG311, a small molecule inhibitor of complex I and hypoxia-induced HIF-1alpha stabilization. Cancer Lett..

[94] Nguyen NNY, Kim SS, Jo YH (2020). Deregulated mitochondrial DNA in diseases. DNA Cell Biol..

[95] Li H, Slone J, Huang T (2020). The role of mitochondrial-related nuclear genes in age-related common disease. Mitochondrion.

[96] Li M, Li G, Yu B, Luo Y, Li Q (2020). Activation of hypoxia-inducible factor-1alpha via succinate dehydrogenase pathway during acute lung injury induced by trauma/hemorrhagic shock. Shock.

[97] Reichard A, Asosingh K (2019). The role of mitochondria in angiogenesis. Mol. Biol. Rep..

[98] Chowdhury AR (2020). Mitochondria-targeted paraquat and metformin mediate ROS production to induce multiple pathways of retrograde signaling: a dose-dependent phenomenon. Redox Biol..

[99] Lee S, Hallis SP, Jung KA, Ryu D, Kwak MK (2019). Impairment of HIF-1alpha-mediated metabolic adaption by NRF2-silencing in breast cancer cells. Redox Biol..

[100] Sharma P, Sampath H (2019). Mitochondrial DNA integrity: role in health and disease. Cells.

[101] Lee KM (2017). MYC and MCL1 cooperatively promote chemotherapy-resistant breast cancer stem cells via regulation of mitochondrial oxidative phosphorylation. Cell Metab..

[102] Oh E-T, Kim CW, Kim HG, Lee J-S, Park HJ (2017). Brusatol-mediated inhibition of c-Myc increases HIF-1α degradation and causes cell death in colorectal cancer under hypoxia. Theranostics.

[103] Hu Q (2019). UHRF1 promotes aerobic glycolysis and proliferation via suppression of SIRT4 in pancreatic cancer. Cancer Lett..

[104] Valle-Mendiola A, Soto-Cruz I (2020). Energy metabolism in cancer: the roles of STAT3 and STAT5 in the regulation of metabolism-related genes. Cancers.

[105] Briston T (2018). VHL-mediated regulation of CHCHD4 and mitochondrial function. Front. Oncol..

[106] Silva-Pavez E, Tapia JC (2020). Protein kinase CK2 in cancer energetics. Front. Oncol..

[107] Liao CP (2018). Loss of MAOA in epithelia inhibits adenocarcinoma development, cell proliferation and cancer stem cells in prostate. Oncogene.

[108] Jezek J, Plecita-Hlavata L, Jezek P (2018). Aglycemic HepG2 cells switch from aminotransferase glutaminolytic pathway of pyruvate utilization to complete Krebs cycle at hypoxia. Front. Endocrinol..

[109] Ramazani M (2019). Analysis of apoptosis related genes in nurses exposed to anti-neoplastic drugs. BMC Pharmacol. Toxicol..

[110] Schito L, Rey S (2018). Cell-autonomous metabolic reprogramming in hypoxia. Trends Cell Biol..

[111] Ye J (2014). Serine catabolism regulates mitochondrial redox control during hypoxia. Cancer Discov..

[112] Samanta D (2016). PHGDH expression is required for mitochondrial redox homeostasis, breast cancer stem cell maintenance, and lung metastasis. Cancer Res..

[113] Nakada C (2020). A transgenic mouse expressing miR-210 in proximal tubule cells shows mitochondrial alteration: possible association of miR-210 with a shift in energy metabolism. J. Pathol..

[114] Lai RK (2016). NDUFA4L2 fine-tunes oxidative stress in hepatocellular carcinoma. Clin. Cancer Res..

[115] Concolino A (2018). Proteomics analysis to assess the role of mitochondria in BRCA1-mediated breast tumorigenesis. Proteomes.

[116] Kung-Chun Chiu D (2019). Hypoxia regulates the mitochondrial activity of hepatocellular carcinoma cells through HIF/HEY1/PINK1 pathway. Cell Death Dis..

[117] Pahima H (2018). Hypoxic-induced truncation of voltage-dependent anion channel 1 is mediated by both asparagine endopeptidase and calpain 1 activities. Oncotarget.

[118] Pan L, Zhou L, Yin W, Bai J, Liu R (2018). miR-125a induces apoptosis, metabolism disorder and migrationimpairment in pancreatic cancer cells by targeting Mfn2-related mitochondrial fission. Int. J. Oncol..

[119] Garcia-Aguilar A, Martinez-Reyes I, Cuezva JM (2019). Changes in the turnover of the cellular proteome during metabolic reprogramming: a role for mtROS in proteostasis. J. Proteome Res..

[120] Yang X (2018). Hypoxia-induced autophagy promotes gemcitabine resistance in human bladder cancer cells through hypoxia-inducible factor 1alpha activation. Int. J. Oncol..

[121] Fallah J, Rini BI (2019). HIF inhibitors: status of current clinical development. Curr. Oncol. Rep..

[122] Zhao XL, Yu CZ (2018). Vosaroxin induces mitochondrial dysfunction and apoptosis in cervical cancer HeLa cells: involvement of AMPK/Sirt3/HIF-1 pathway. Chem. Biol. Interact..

[123] Jin J (2019). Cardamonin inhibits breast cancer growth by repressing HIF-1alpha-dependent metabolic reprogramming. J. Exp. Clin. Cancer Res..

[124] Xia Y, Jiang L, Zhong T (2018). The role of HIF-1alpha in chemo-/radioresistant tumors. OncoTargets Ther..

[125] Nagaraju GP (2019). Inhibition of HSP90 overcomes resistance to chemotherapy and radiotherapy in pancreatic cancer. Int. J. Cancer.

[126] Wang D, Qin Q, Jiang QJ, Wang DF (2016). Bortezomib sensitizes esophageal squamous cancer cells to radiotherapy by suppressing the expression of HIF-1alpha and apoptosis proteins. J. X-ray Sci. Technol..

[127] Cheng Y, Ji Y (2020). Mitochondria-targeting nanomedicine self-assembled from GSH-responsive paclitaxel-ss-berberine conjugate for synergetic cancer treatment with enhanced cytotoxicity. J. Control. Rel..

[128] Djeungoue-Petga M-A (2019). Intramitochondrial Src kinase links mitochondrial dysfunctions and aggressiveness of breast cancer cells. Cell Death Dis..

[129] Kinnaird A (2016). Metabolic modulation of clear-cell renal cell carcinoma with dichloroacetate, an inhibitor of pyruvate dehydrogenase kinase. Eur. Urol..

[130] Jung N, Kwon HJ, Jung HJ (2018). Downregulation of mitochondrial UQCRB inhibits cancer stem cell-like properties in glioblastoma. Int. J. Oncol..

